# Pan-Genome Analyses of *Geobacillus* spp. Reveal Genetic Characteristics and Composting Potential

**DOI:** 10.3390/ijms21093393

**Published:** 2020-05-11

**Authors:** Mengmeng Wang, Han Zhu, Zhijian Kong, Tuo Li, Lei Ma, Dongyang Liu, Qirong Shen

**Affiliations:** Jiangsu Provincial Key Lab of Solid Organic Waste Utilization, Jiangsu Collaborative Innovation Center of Solid Organic Wastes, Educational Ministry Engineering Center of Resource-Saving Fertilizers, Nanjing Agricultural University, Nanjing 210095, China; 2013203040@njau.edu.cn (M.W.); 2018103120@njau.edu.cn (H.Z.); 2018203038@njau.edu.cn (Z.K.); 2018203039@njau.edu.cn (T.L.); 2016203031@njau.edu.cn (L.M.); shenqirong@njau.edu.cn (Q.S.)

**Keywords:** *Geobacillus*, comparative genomics, pan-genome, core genome, genomic features, evolutionary events

## Abstract

The genus *Geobacillus* is abundant in ecological diversity and is also well-known as an authoritative source for producing various thermostable enzymes. Although it is clear now that *Geobacillus* evolved from *Bacillus*, relatively little knowledge has been obtained regarding its evolutionary mechanism, which might also contribute to its ecological diversity and biotechnology potential. Here, a statistical comparison of thirty-two *Geobacillus* genomes was performed with a specific focus on pan- and core genomes. The pan-genome of this set of *Geobacillus* strains contained 14,913 genes, and the core genome contained 940 genes. The Clusters of Orthologous Groups (COG) and Carbohydrate-Active Enzymes (CAZymes) analysis revealed that the *Geobacillus* strains had huge potential industrial application in composting for agricultural waste management. Detailed comparative analyses showed that basic functional classes and housekeeping genes were conserved in the core genome, while genes associated with environmental interaction or energy metabolism were more enriched in the pan-genome. Therefore, the evolution of *Geobacillus* seems to be guided by environmental parameters. In addition, horizontal gene transfer (HGT) events among different *Geobacillus* species were detected. Altogether, pan-genome analysis was a useful method for detecting the evolutionary mechanism, and *Geobacillus*’ evolution was directed by the environment and HGT events.

## 1. Introduction

The genus *Geobacillus* are rod-shaped facultative anaerobic or aerobic thermophilic Gram-positive microbes that seem to be very common in different habitats. Spores of *Geobacillus* can be isolated in high numbers not only from extreme environments such as hydrothermal vents, oil reservoirs, hot springs, and mining environments, but also from typical temperature soils or even cold ocean environments [1]. *Geobacillus* have a reputation in biotechnology for their ability to secrete thermostable enzymes and digest hemicellulose [2], which could contribute to the biodegradation and be a good source for industrial enzymes. Most *Geobacillus* strains can ferment C5 and C6 sugars simultaneously, and various *Geobacillus* strains were reported to have the ability to degrade hemicellulose [3]. Important enzymes originating from *Geobacillus* spp. include glycoside hydrolases [4,5], lipases [6], and protease [7]. Moreover, the recent report indicated that *Geobacillus stearothermophilus* could produce organic acids in organic matter fermentation, thus reducing ammonia emissions during composting [8].

Microbial taxonomy is a means by which organisms are unambiguously assigned to distinct taxa; thus, it ascertains the relatedness among the organisms. Various mature methods were developed in the classification of microorganisms, such as DNA–DNA hybridization (DDH) and 16S rRNA sequence analysis, which mainly relied on the estimation of sequence similarity between organisms [9]. Assignment of some strains to a certain species required the DDH value greater than 70% or the 16S rRNA sequence identity greater than 97% [10]. Furthermore, the *recN* gene has been demonstrated to be more robust for identifying *Geobacillus* strains at the genus and species levels [11]. Latterly, in the light of improvements of sequencing technology, some new approaches were taken in microbial taxonomy, including Average Nucleotide Identity (ANI) and the genome-to-genome distance method (GGDC), which have been demonstrated to be useful in evaluating the relatedness of any microbial genomes [12,13]. *Geobacillus* strains were previously described as belonging to the species *Bacillus stearothermophilus*, but more evidence had gradually revealed that these microbes exhibited a great deal of heterogeneity at optimum living temperatures in terms of physiological and some other phenotypic characteristics. They were subsequently reclassified as the new genus *Geobacillus* [14], which contains diverse species that differ in both genotype and phenotype [15]. Recently, *Geobacillus* strains with GC content ranging from 42.1% to 44.4% were designed as a novel genus *Parageobacillus*, with *P. thermoglucosidasius* as the type species [16].

Over the past decade, genomic data have experienced explosive growth with the rapid reduction of sequencing costs. Therefore, various related approaches have been developed to comprehensively explore metabolic characteristics and obtain evolutionary insights. In particular, the pan-genome analysis has been introduced to not only explain the metabolic diversity of a chosen phylogenetic clade [17], but also explore its classification and evolutionary history [18]. Some terms were also created to describe this variation, such as ‘core’ and ‘accessory’ genomes. The core genome refers to key genes that commonly exist in every member of a specific genome set, and the accessory genome represents dispensable genes which only exist in some of the genomes [19]. The pan-genome refers to all the genes that are detected in the whole group, including the core and the accessory genome [20]. Some prokaryotic species possess genomes with high similarity in gene content (closed pan-genomes), while others possess open pan-genomes. The status of a pan-genome is related to the number of the datasets involved in the analysis, as well as the characteristics of the species, such as the ability to integrate exogenous DNA and living environment of the species [21]. HGT might contribute to the variability of genome content within prokaryotes, allied with differential gene losses, which are closely associated with microbial adaptation and evolution [22]. Vast amounts of prokaryotic genome data now efficiently reveal the pervasive effect of introgressions within various species [23].

Although the significant characteristics and evolutionary relationships of *Bacillus* species have been fully explored, few works have been performed on the genus *Geobacillus*. Besides that, the genomic features within *Geobacillus* have not been comprehensively studied according to the pan-genome information of this group. In this study, pan-genome analysis was used to understand the evolutionary relationships and metabolic features for *Geobacillus* more comprehensively. This work conveys exciting and unique findings to the readers and provides insights into the evolution mechanism, gene exchange, and the biodegradation potential of *Geobacillus* group members.

## 2. Results

### 2.1. Pan- and Core Genomes Analysis of Different Geobacillus Species

The pan- and core genomes development plots of the *G. stearothermophilus*, *G. thermocatenulatus*, *G. thermodenitrificans*, and *P. thermoglucosidasius* species displayed similar evolution (Supplementary Materials Figure S1). The four species possess open pan-genomes that continuously grow as new genomes are added. *G. stearothermophilus* exhibited 5899 genes after the inclusion of eight genomes, while 7115, 5191, and 6748 genes were found for *G. thermocatenulatus*, *G. thermodenitrificans*, and *P. thermoglucosidasius*, respectively (Table 1). The growth exponent value was 0.283 for *G. stearothermophilus*, versus 0.216, 0.175, and 0.253 for *G. thermocatenulatus*, *G. thermodenitrificans*, and *P. thermoglucosidasius*, respectively. The core genome trends were also similar, declining rapidly to final totals of 1994, 2142, 2861, and 2659 genes. These results were caused mainly by the different species and their living habitat because the pan- and core genomes represented the functions of the whole genomes participating in the pan-genome analysis.

### 2.2. COG Distribution Between Different Geobacillus Species

**Figure 1:**
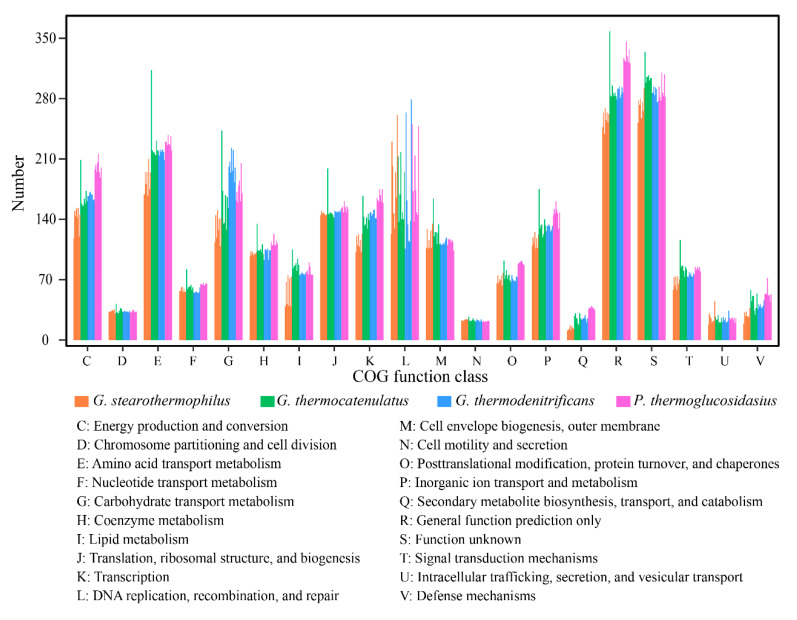
Comparisons of COG functional categories of *G. stearothermophilus*, *G. thermocatenulatus*, *G. thermodenitrificans*, and *P. thermoglucosidasius*. For each category, from left to right are strain ATCC 12980 ^T^, B5, Sah69, 53, B4109, C1BS50MT1, D1, DSM 458, BCO2, GS-1, DSM 730^T^, KCTC3921, SURF-114, SURF-48B, SURF-189, T6, DSM 465 ^T^, ID-1, NG80-2, PA-3, JSC-T9a, KCTC3902, OS27, T12, C56-YS93, DSM 2542 ^T^, GT23, ZCTH02-B4, TG4, TNO-09.020, W-2, and Y4.1MC1.

To compare the functional distribution between the four species, categories of COG were analyzed (Figure 1). Generally, about 57% of the genes were annotated by the COG database, and the functional distribution within each species was similar among most categories. As expected, the most abundant functional groups were general function unknown (S) or function prediction only (R). Aside from those two non-deterministic groups, the largest COG group was the amino acid transport and metabolism (E), followed by replication, recombination and repair (L), and energy production and conversion (C), whose average genes were 215, 175, and 167, respectively. Some COG groups number were quite close among the four species, like cell cycle control, cell division, chromosome partitioning (D), nucleotide transport and metabolism (F), coenzyme transport and metabolism (H), translation, ribosomal structure and biogenesis (J), cell wall/membrane/envelope biogenesis (M), cell motility (N) and intracellular trafficking, secretion, and vesicular transport (U). However, some COG groups fluctuated greatly even within same species, like replication, recombination and repair (L), and carbohydrate transport and metabolism (G). In addition, the COG function genes of *P. thermoglucosidasius* strains were more abundant than that of the other species, especially in the categories of energy production and conversion (C), general function prediction only (R), and defense mechanism (V). The different abundance of function genes between species might reflect their adaptability to the environment. *P. thermoglucosidasius* encoded more function genes related to energy metabolism and defense system, thus it might be more open during the evolution history.

### 2.3. CAZyme Analysis of Different Geobacillus Species

**Figure 2:**
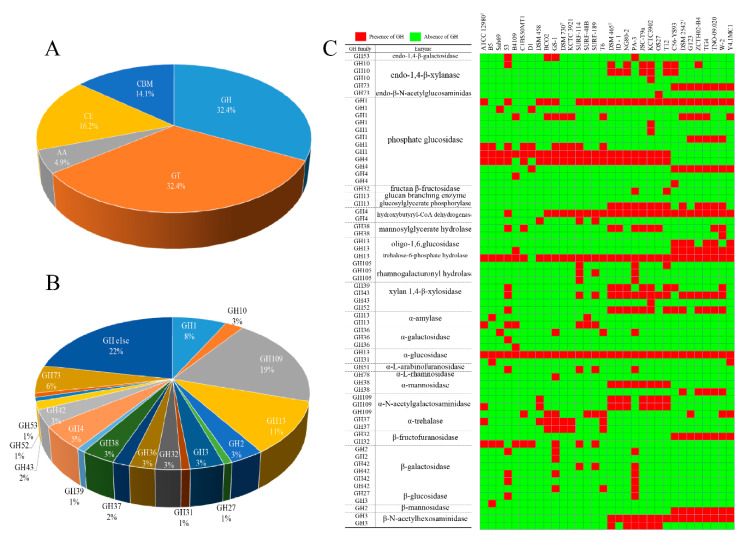
GH family distribution of all genomes. (**A**) Pie chart indicating the percentage of each category of CAZymes. (**B**) Pie chart indicating the percentage of each GH family. (**C**) Heatmap displaying the GH family members identified in all the genomes.

The genus *Geobacillus* are capable of producing kinds of thermophilic enzymes. In order to understand the fermentation capabilities of *Geobacillus* in solid condition of composting ecosystem, the pan-genome were BLAST against the CAZyme database, to screen genes that catalyze the hydrolysis of plant polymers. The *Geobacillus* pan-genome contained 364 different CAZymes genes distributed unequally among glycosyl transferases (GT, 32.4%), glycoside hydrolases (GH, 32.4%), auxiliary activities (AA, 4.9%), carbohydrate esterases (CE, 16.2%), and carbohydrate-binding modules (CBM, 14.1%) (Figure 2A). For the GH family, the analysis showed that the *Geobacillus* strains encoded genes that belong to 21 different GH families (Figure 2B and Supplementary Table S3). Distributed across different strains, GH members were found to be putatively assigned to degrading starch, hemicellulose, and other oligosaccharides, classified as amylase (GH13), xylanase (GH10), and 1,4-β-xylosidase (GH39, GH43, and GH52). The result also revealed enzymes with predicted α-mannosidase (GH38), β-mannosidase (GH2), β-galactosidase (GH2, GH42, GH53), β-galactosidase (GH36), fructan β-fructosidase (GH1), and, though at a lower abundance, α-trehalase (GH37), β-fructofuranosidase (GH32), α-L-arabinofuranosidase (GH51), and α-L-rhamnosidase (GH78) (Figure 2C). The GH family members from prediction allowed us to identify certain GHs that appeared to be characteristic of *Geobacillus* strains. In addition, given the phylogenetic analysis, *G. thermodenitrificans* and *P. thermoglucosidasius* tended to encode more GH family genes, especially in GH3, GH13, and GH109 families.

### 2.4. Calculation of Pan- and Core Genomes of All Strains

**Figure 3:**
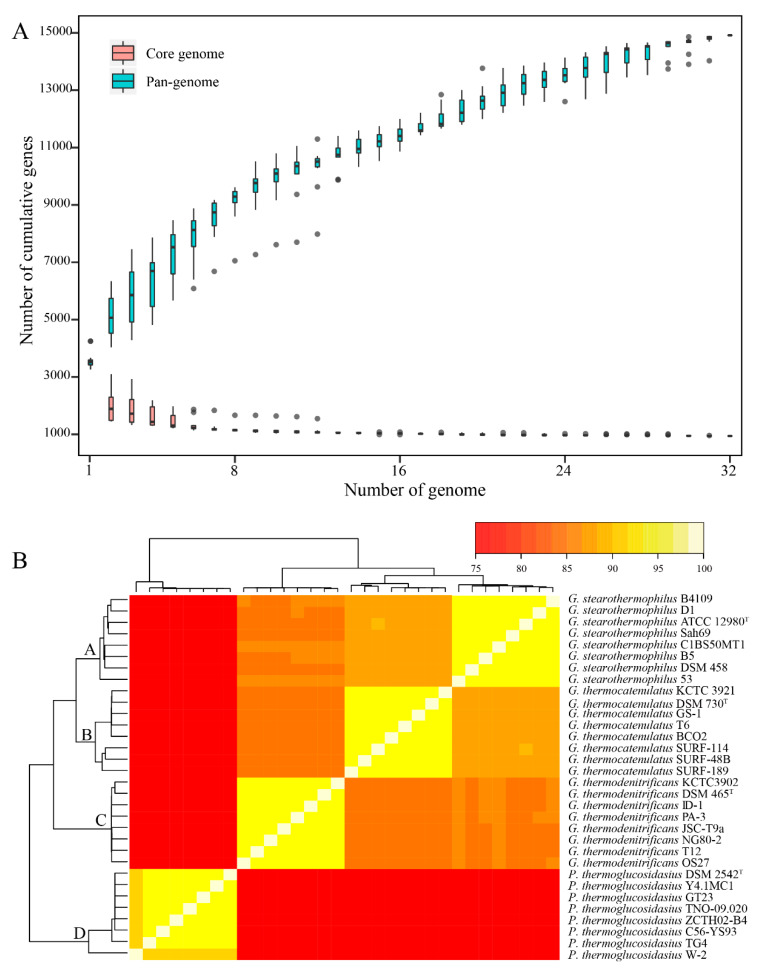
The pan-genome and clustering analysis of *Geobacillus* strains. (**A**) Development plots of the pan- and core genomes. The boxplots of the pan- (cyan color) and core genomes (pink color) are plotted. (**B**) Clustering of the *Geobacillus* strains based on the pan-genome ANI matrix. (A) *G. stearothermophilus* clade, (B) *G. thermocatenulatus* clade, (C) *G. thermodenitrificans* clade, and (D) *P. thermoglucosidasius* clade.

To get a general view of the genomic contents, the pan- and core genomes of the 32 genomes were calculated and visualized (Figure 3A). The number of pan-genome genes was still increasing, with approximately 152 genes per genome when the last genome was added to the calculation; hence, the pan-genome could be regarded as open. The number of core genome genes decreased rapidly with the addition of genomes but stabilized slowly after the 12th genome was added, indicating that the core genome was closed. The core genome contained 940 genes, and the pan-genome contained 14,913 genes. A clustering based on the ANI of the pan-genome revealed four significant clades (Figure 3B): (A) *G. stearothermophilus* clade, (B) *G. thermocatenulatus* clade, (C) *G. thermodenitrificans* clade, and (D) *P. thermoglucosidasius* clade. The ANI matrix is stored in Supplementary Table S4. As expected, same species clustered together, and the ANI within same species are around 95%–96%. However, the ANI between *P. thermoglucosidasius* W-2 and other *P. thermoglucosidasius* strains is about 92.17%, slightly lower than the cutoff score 95% of defining same species [24]. It might be caused by the low-quality sequence of the draft genome [25]. For *Geobacillus* strains, the *recN* gene is a more robust marker for identifying a new strain at the genus and species levels [11]. This result is highly consistent with the phylogenetic tree built by *recN* genes. In addition, a clustering based on the ANI of the core genome was also carried out (Supplementary Figure S2), and the four species clustered clearly within each clade. The high similarity of the pan- and core genome clusters revealed that the evolutionary relationship already appeared in the core genome.

### 2.5. KEGG Analysis of Geobacillus Strains

The metabolic capacity of the pan- and core genomes of *Geobacillus* strains was evaluated by the online Brite mapping tool and GhostKOALA pathway reconstruction tool (Table 2). Brite mapping revealed an average extension of 2-fold in the pan-genome compared with the core genome. A greater gene extension of 2.8-fold was observed in the pan-genome for the class “signaling and cellular processes”, and a relatively lower extension of 1.4-fold was detected in “genetic information processing”. The result of pathway reconstruction showed an average of 1.8-fold extension in the pan-genome compared with the core genome, within which a clearly greater extension of 3.5-fold was observed for “environmental information processing”, a relatively lower extension of 1.2-fold for “genetic information processing”, similar with the observations in Brite mapping analysis.

**Figure 4:**
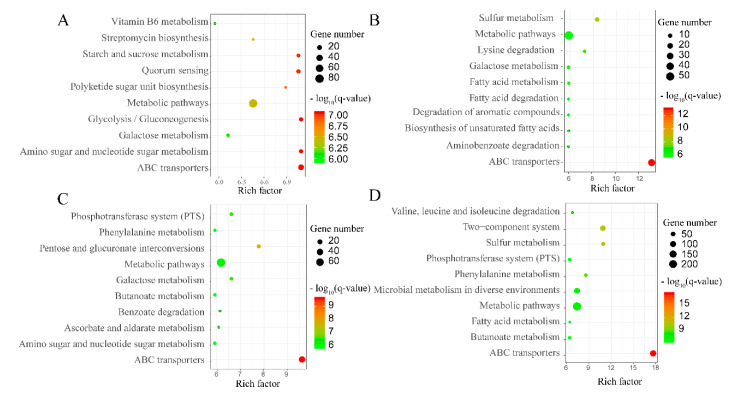
KEGG pathway enrichment scatter plot of specific genes for different *Geobacillus* species. The *x*-axis represents the rich factor, and the *y*-axis shows the name of the KEGG pathway. Dot size represents the number of associated genes, and the color indicates the −log_10_(q-value). (**A**–**D**) Representations of the enrichment analysis of *G. stearothermophilus*, *G. thermocatenulatus*, *G. thermodenitrificans*, and *P. thermoglucosidasius*, respectively.

The results of module reconstruction against the KEGG reference module indicated that short pathways were usually complete in the core genome, while a lack of one or two enzymes usually occurred in longer pathways (Supplementary Table S5). Some long pathways, such as the fatty acid metabolism, phosphatidyl ethanolamine synthesis, F-type ATPase, cysteine synthesis, and phosphate transport protein pathways, were complete in the softcore genome. In addition, metabolic pathways, especially the ones associated with carbohydrate and some amino acid metabolism, were mostly complete in the core genome, while pathways related to some co-factors, regulatory systems, and sugar-, polyol-, and lipid-related transport systems were mostly deficient. Moreover, most of these genes were related to environmental interactions. To investigate the specificity of different *Geobacillus* species, an KEGG enrichment analysis was carried out according to the specific accessory genome of each species, and the most significant KEGG pathway is shown in Figure 4. The results indicated that most pathways were specifically enriched in one species; thus, different species have evolved a specific metabolism during their long history of mutation. However, there were also some same pathways enriched in more than one species, like ABC transporters, metabolic pathways, galactose metabolism, and fatty acid metabolism. These might be caused by the existence of isozymes.

### 2.6. Analyses of SNPs, Representative Genes, and HGT Events

To determine whether SNPs were related to the phylogenic relationships of *Geobacillus*, calculations were performed for 940 core genes in the 32 genomes, and 12,983 SNPs were detected in total. SNPs existed widely in *Geobacillus*, and their existence contributed to the diversity of the *Geobacillus* strains. In addition, to determine which genes built more similar phylogenetic trees, the pan-genome tree was set as a reference for comparison with all trees built for genes in the core genome. The distances between different trees were calculated, and 40 genes were finally selected (Supplementary Table S6). These 40 genes exhibited an average length of 1302 bp, among which 15 were interacting with RNA or DNA, and there were also some hypothetical genes detected. The average length of the other core genes was 871 bp. It seems that longer genes might possess more genomic information and were more suitable for representing the phylogenetic relationships at the genus level. Previously, we thought that the SNP density of the 40 genes might be lower than other core genomes, but the truth is that there was no significant difference among them. These genes are neither higher nor lower homologous than the others, and the features of the representative genes reflected more in their length.

**Figure 5:**
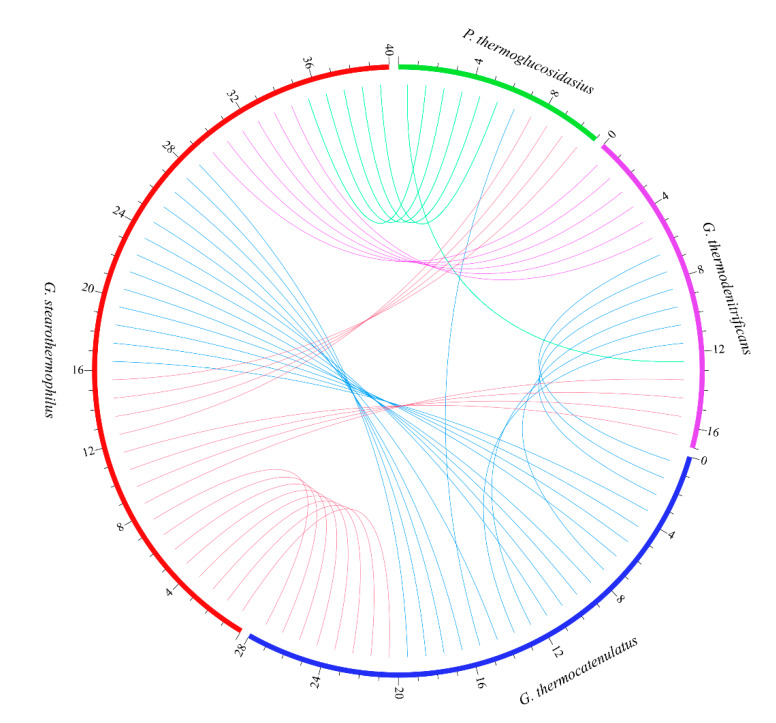
The predicted horizontal gene transfer (HGT) events between different species. Bands connect the two species where HGT events occurred, and the outmost digits are the HGT events numbers within each species.

In order to predict whether any of the specific genes in the four groups of *Geobacillus* participated in the HGT events, the genomes of all strains were interrogated by MetaCHIP pipeline, and 48 different genes were considered to be potential transferred genes, based on the analysis results. Among them, specific genes, including transposases, phage related proteins, hypothetical proteins, ABC transporter, and transcriptional regulators, constituted a large proportion (Supplementary Table S7). In general, transposases, phage-related proteins, and some hypothetical proteins might commonly be associated with HGT events, while ABC transporter and transcriptional regulators were discovered to have participated in DNA transfer or transformation regulation recently. Figure 5 exhibited the possible HGT events between different species, and the results showed that *G. stearothermophilus* could be the most active species involved in the HGT process.

## 3. Discussion

*Geobacillus* strains are abundant in their genetic, ecological, and physiological diversity. However, it is still unclear how the diverse environments influence their genome contents and how this change may affect their lifestyles in turn. Here, a comparative analysis was conducted, with 32 *Geobacillus* genomes derived from geographically distinct origins. Pan-genome analysis is a critical approach for exploring the genomic and metabolic repertoires of a phylogenetic lineage in microbes [26]. As more genomes are added in the dataset, the number of core genome genes tends to decrease, while the number of pan-genome genes tends to increase [27], and continuous genome addition will change these numbers. The pan-genomes of the *Geobacillus* species showed increases with exponent values of approximately 0.2, suggesting that it was open, experiencing frequent evolutionary changes through gene gains and losses or lateral gene transfers for efficient environmental adaptations. The open feature was consistent with the analysis from different dataset [28] and was opposite to other species, such as *Bifidobacterium breve* and *Staphylococcus lugdunensis*, which showed an essentially closed trend [18,29]. Clustering based on the pan- and core genome ANI was carried out, and only a slight difference appeared between them, indicating that the evolutionary relationship was clear in both pan- and core genomes. Previously, Burgess, et al. [30] conducted a detailed comparative genomic analysis within 63 *Geobacillus* strains, and they considered that the core genome was more suitable for phylogeny analysis than the pan-genome ANI because the pan-genome contained many accessory genes acquired via HGT that might reduce the resolution of phylogeny. Besides, the author put forward that combining genomic data with phenotypic data for defining a bacterial species was not suitable for the *Geobacillus* taxon, because the phenotype within same species varied. It is a pity that these results cannot be further supported in this research, for we have no phenotypic data for verification, and the phylogenic relationship based on the core and pan-genome ANI shows a slight difference here. It might be caused by the dataset in our analysis containing a lower number of species; thus, the high resolution of core genome was ignored. In our study, the effects of HGT events were highlighted. HGT might be the power that drives the evolution of *Geobacillus* from *Bacillus*, which also contribute to the ecological diversity and gene abundance, according to the COG function categories, CAZymes and KEGG analysis. Furthermore, *Geobacillus* strains were analyzed combined, with the phylogeny, and our results indicated that *G. stearothermophilus* and *P. thermoglucosidasius* tend to be more open, while *G. thermodenitrificans* and *P. thermoglucosidasius* encoded more abundant hydrolase genes and, thus, owned better application potential in bioenergy or composting industry.

The *Geobacillus* pan-genome contained various genes related to the metabolisms of the glucose, xylose, mannose, and galactose of COG category G (Supplementary Table S2), indicating that the plant polysaccharides containing glucan-, xylan-, and arabinose-structures could be consumed by *Geobacillus* strains. In addition, genes associated with polysaccharide transport and phosphotransferase systems were as numerous as those of polysaccharide-degrading enzymes. For example, the ABC-type sugar transporters COG1129, COG0395, and COG1175 showed high frequency in the pan-genome, and these transporters were mainly related to the uptake of carbohydrate [31]. Another rich function related to carbohydrate was the arabinose efflux permease family (COG2814), which is associated with the import and export of compounds, including sugars, amino acids, and antimicrobials [32]. Moreover, we also detected abundant phosphotransferase system genes (COG1263, COG 1762, and COG1925). They were reported to play a role in the phosphorylation and transport of various monosaccharides, disaccharides, polyols, amino sugars, and other sugar derivatives [33]. In other words, the wide distribution of COGs related to carbohydrate metabolism and transport of *Geobacillus* reveals a good potential for sugar transport and hemicellulose degradation in composting.

*Geobacillus* strains are well-known for their capacities to degrade hemicellulose polymers, and the orthologous hemicellulose utilization loci were identified in most *Geobacillus* strains [34]. In our study, the analysis results provided detailed genetic evidence for both hydrolysis and transglycosylatic abilities of this genus. The GH family contains a mass of enzymes which can hydrolyze the glycosidic bond between carbohydrates. The abundant hemicellulose-degrading enzymes detected in our pan-genome suggested that the *Geobacillus* strains mainly participated in metabolizing partially degraded plant materials during composting. Starch and hemicellulose degrading enzymes were present in GH27, GH3, GH31, GH10, and GH39 mainly, and these enzymes were reported to convert complex polysaccharides into mono- and oligosaccharides, which could then be taken up by ABC-type transporters for further internal hydrolysis and metabolism. Oligosaccharide-degrading enzymes related to the processing of small oligosaccharides were most found in GH2, GH3, and GH13. Furthermore, genes encoding α-amylase were widely detected in the *Geobacillus* strain, which was considered to be a crucial amylase. *Geobacillus* strains showed a significantly higher number of GH13 family genes, and they mainly participated in hydrolyzing starch [35]. Of all the GH family members, GH13 and GH109 accounted for the largest slice, and they were known as the dehydrogenase and α-amylase family associated with starch biosynthesis and turnover [36]. The members of GH23 family were also abundant in *Geobacillus*, and this family was thought to harbor lytic transglycosylases and inverting lysozymes, which can cleave glycosidic bonds by a substrate-assisted mechanism [37]. The GH1 members encoded by *Geobacillus* were 6-phospho-β-glycosidases, which were highly thermostable and remained most activity after incubation at 60 °C for seven days, and they could utilize cellobiose [5]. Most of the *Geobacillus* strains also encoded arabinofuranosidases (GH51) and acetyl xylan esterases, and those enzymes could simultaneously hydrolyze the arabinofuranosyl linkages in arabinoxylan and the acetyl substitutions on xylose moieties, respectively [38]. Considering the synergisms between xylanases and arabinofuranosidases/esterases in the enzymatic degradation of xylan, we suggest that arabinofuranosidase and esterases may greatly facilitate the breakdown of xylan during the conversion of hemicellulose. Furthermore, De Maayer, Brumm, Mead and Cowan [34] revealed that the hemicellulose utilization locus was widespread in *Geobacillus* strains and implied that it had a highly variable ability of degrading distinct hemicellulose substrates. The abundant GH family genes in the pan-genome of *Geobacillus* indicated that abundant enzymes participated in hydrolyzing the glycosidic bond between two carbohydrates, or between carbohydrate and non-carbohydrate moiety, thus resulting in a strong ability for biomass degradation. In addition to the glucoside hydrolases, we detected 34 CBM50 genes, which accounted for 66.7% of all CBM family members in *Geobacillus* pan-genome. CBMs are sugar-binding proteins which consist of contiguous amino acid sequence within a larger encoded protein sequence. The roles of CBM proteins was to enhance the catalytic efficiency of the multi-modular carbohydrate-active enzyme by binding to the carbohydrate ligand [39]. It is rational that the abundance of CBM50, along with GH23, leads to the efficiency of degrading insoluble hemicellulosic plant biomass for *Geobacillus* during composting. Together with the COG analysis of hemicellulose degradation, the rich repertoire of CAZymes provides the basic which can support the process and metabolism of carbohydrates involved in the composting ecosystem. Furthermore, the CAZyme genes were mostly distributed in the accessory genome rather than the core genome, highlighting the importance of HGT and its contribution to the diversity of the metabolic machinery and, consequently, to their ecological importance and biotechnological potential.

The genomes of *Geobacillus* strains are open; thus, the pan-genomes are abundant and could be gained or lost in high frequency. The analysis of functional categories enriched in a pan-genome might provide valuable information for evolutionary developments in a bacterial lineage [40]. In the Brite mapping analysis, genes related to housekeeping processes, such as genetic information processing, were least overrepresented in the pan-genome, while those genes involved in signaling and cellular processing were the most overrepresented. Similar results were observed in the analysis of pathway reconstruction and module reconstruction. Taken together, the results indicated that genes related to fundamental process in the cells were mostly conserved in evolution and usually did not change with the environment. However, genes that interacted with the environment were often obtained through environmental changes and were widely present in the pan-genome. Those results were similar with the analysis of COG distributions in the previous study [28]. Moreover, in order to investigate the specificity of different *Geobacillus* species, an KEGG enrichment analysis was carried out according to the accessory genome of each species. Some same KEGG pathways were enriched in more than one species, such as ABC transporters, metabolic pathways, galactose metabolism, and fatty acid metabolism, which belonged to huge and fundamental pathways. For those pathways, most genes were conserved in all species, but still some differences existed that were caused by the mutation during the long evolution process. Specifically, the accessory genes in *G. stearothermophilus* were more enriched in carbohydrate metabolism pathways, such as the starch and sucrose metabolism pathway, amino sugar and nucleotide sugar metabolism pathway, and glycolysis/gluconeogenesis pathway. It even had abundant genes for the vitamin B6 metabolism pathway. *G. stearothermophilus* was the type species of the genus *Geobacillus*, and it was widely distributed and readily isolated from natural or manmade biotopes. In our dataset, the eight *G. stearothermophilus* strains even came from eight different habitats. The rich carbohydrate metabolism pathways, along with other co-pathways, formed the basis of adaption for various environmental factors. Furthermore, it seems that *P. thermoglucosidasius* strains encoded more abundant metabolism pathways, including butanoate metabolism, valine/leucine/isoleucine degradation, and microbial metabolism in diverse environments. *P. thermoglucosidasius* used mixed-acid fermentation in anaerobic conditions to produce lactate, ethanol acetate, and carbon dioxide [41], and the abundant microbial metabolism pathways made it more feasible. The overrepresentation of the functional genes in the pan-genome related to the core genome also indicated the importance of HGT and its contribution toward the ecological and metabolic diversity in *Geobacillus* species.

The genes building most similar phylogenetic trees with the pan-genome tree were selected, among which 15 were associated with rRNA or DNA, like encoding rRNA subunit or repairing DNA. The 16S rRNA sequence was always taken to identify bacteria, while the 18S rRNA sequence was used to identify fungi. We also detected the *recN* gene, as expected, which proved to be the most robust marker for the identification of the genus *Geobacillus* [11]. In addition, genes of *infB*, *rpoB*, and *spo0A* were also on the list, and they have been shown to be useful in the identification of *Geobacillus* [42,43,44]. The SNP density of the 40 genes are not special when compared with other core genomes, but the 40 genes possessed a clear longer gene length, which might mean that longer genes kept more genomic information and were more suitable for representing the phylogenetic relationships at the species level.

## 4. Materials and Methods

### 4.1. Genome Sequences

The genomes included in the comparative analyses were acquired from the NCBI and PATRIC database, comprising *G. stearothermophilus*, *G. thermocatenulatus*, *G. thermodenitrificans*, and *P. thermoglucosidasius*. The primary features of all genomes were described in Supplementary Table S1.

### 4.2. Calculation of Pan- and Core Genomes

All the genomes were previously re-annotated combined by Prodigal [45] and Genemark [46] software. If the prediction results of the same location were different, the result predicted by Prodigal would be preferred. The pan- and core genomes were calculated with GET_HOMOLOGUES software [47], and the OrthoMCL algorithm [48] was taken to generate orthologous clusters. The phylogenetic tree of *Geobacillus* was built according to the ANI value matrix. In a pan-genome analysis, the number of accumulated genes related to the number of genomes can be expected by Heaps’ law (n = k × *N*^−α^, where *N* is the number of participated strains, n is the number of estimated genes, and k is a constant to fit the curve) [49]. According to the Heaps’ law, the pan-genome is open when α < 1, in which case each new participated genome brings new genes and, hence, continuously extends the total set of genes, whereas α > 1 indicates a closed pan-genome, which means a small number of genomes already cover the complete gene contents, so that new genomes do not contribute to the gene content significantly.

The four clusters based on the pan-genome analyses were defined as previously described by Kaas, et al. [50]. The core genome is a set of genes that are shared by all genomes; softcore genes refer to genes that exist in 95–100% of the dataset; shell genes refer to genes that exist in more than two but less than 95% of the dataset; cloud genes exist in no more than two of the genomes. The developing plot of the pan- and core genomes was initiated by calculating orthologous genes and adding one single genome each time, until all genomes were included. The order of the comparison list was randomized ten times, and the results were saved in a matrix, for later analysis.

### 4.3. Functional Classification of COGs and Identification of CAZymes

All genomes were annotated by BLAST searches against the COG database [51], with the E-value to 10^−5^, and then categorized into different subgroups, separately. The COG annotation results were merged into one file, according to the pan-genome below (Supplementary Table S2). The dbCAN database [52] was used to generate hidden Markov models (HMMs) [53], followed by mapping against the CAZyme database [54]. The results were later processed to confirm the distributions of different GH families.

### 4.4. Clustering and GhostKOALA Analysis of the Pan-Genomes

Pan-, core, and softcore genomes were annotated by using Kyoto Encyclopedia of Genes and Genomes (KEGG) internal tool GhostKOALA via BLAST searches against the “genus_prokaryotes + family_eukaryotes” database with the Brite, Pathway, and Module reconstruction algorithms [55]. The mapping numbers of each category were counted for pan-, core, and softcore genome separately. Then the core and softcore genome number were divided by the pan-genome number, thereby getting the extension. Brite mapping captured the functional hierarchies of different biological KEGG objects. Pathway reconstruction search against all pathways of KEGG and Module reconstruction was performed by using sets of K numbers against the KEGG reference module, to evaluate whether a block was complete or not. To investigate the specificity of different *Geobacillus* species, the accessory genome of each species was extracted, followed by an KEGG enrichment analysis.

### 4.5. Identification of Representative Genes and HGT Events

To identify which genes represented the phylogenetic relationship of *Geobacillus* most closely, the tree built by each core genome was compared with the pan-genome phylogenetic tree. Briefly, each homologous gene was extracted as a single FASTA file and then transformed to PHYLIP format with the python script fasta_to_phylip. The PHYLIP files were imported into RaxML software [56], and PHYLIP trees were constructed by using the ‘neighbor-joining’ method, with a Bootstrap setting of 1000. The tree of each core genome is shown in NEXUS format and compared to the reference tree with TreeCmp software [57]. The metric options chosen were the matching split and the quartet distance [58,59], and the top 5% of genes building the most similar phylogenetic trees to the pan-genome tree were selected. To analyze the SNP distribution, the homologous gene sets of the core genome were imported into MATLAB (R2018b), to determine the consensus sequences. All the genes were compared with the consensus sequences, and SNPs were identified according to their specificity in *Geobacillus*. The regions of HGT events were detected according to the software MetaCHIP [60].

## 5. Conclusions

Complete genome-based pan- and core genomes studies of *Geobacillus* are useful for defining and distinguishing the roles of functional genes and how they may contribute to the diversity of this genus. The analysis of COG and CAZymes exhibited considerable potential industrial applications in agricultural wastes management. KEGG reconstruction results indicated that most functional genes overrepresented in the pan-genome were involved in adaptation to the environment. Combined with the HGT events detected above, the evidence about the gene exchange between different species was already clear. Thus, *Geobacillus* strains were considered as nomadic, and HGT has played a vital role in their evolution.

## Figures and Tables

**Table 1:** Genomic statistics and pan-genome information of all species.

Species	N_genome_	Size (Mb)	N_genes_	N_core_	N_shell_	N_cloud_	N_pan_
-	32	3.54 ± 0.35	3629 ± 356	940	5304	8496	14913
*G. stearothermophilus*	8	3.13 ± 0.33	3280 ± 281	1994	1925	1980	5899
*G. thermocatenulatus*	8	3.53 ± 0.11	3794 ± 360	2142	2333	2640	7115
*G. thermodenitrificans*	8	3.58 ± 0.13	3532 ± 153	2861	1101	1229	5191
*P. thermoglucosidasius*	8	3.92 ± 0.19	3912 ± 192	2659	1737	2352	6748

**Table 2:** Reconstruction of pan-, core, and softcore genome of all the genomes with Brite and Pathway algorithm of GhostKOALA.

	Gene Numbers			n-Fold Extension	
	Pan	Softcore	Core	Softcore-Pan	Core-Pan
Brite mapping					
Orthologs and modules	2846	1625	1419	1.8	2.0
Protein families: metabolism	1342	798	698	1.7	1.9
Protein families: genetic information processing	516	399	372	1.3	1.4
Protein families: signaling and cellular processing	539	228	190	2.4	2.8
Total	5243	3050	2679	1.7	2.0
Pathway reconstruction					
Metabolism	2958	1850	1666	1.6	1.8
Genetic Information Processing	198	180	167	1.1	1.2
Environmental Information Processing	278	98	79	2.8	3.5
Cellular Processes	156	102	78	1.5	2.0
Organismal Systems	61	30	27	2.0	2.3
Human Diseases	88	49	47	1.8	1.9
Total	3739	2309	2064	1.6	1.8

## Supplementary Materials

Supplementary materials can be found at https://www.mdpi.com/1422-0067/21/9/3393/s1.

## Abbreviations

COG	Clusters of orthologous groups
CAZymes	Carbohydrate-active enzymes
HGT	Horizontal gene transfer
KEGG	Kyoto Encyclopedia of Genes and Genomes
ANI	Average nucleotide sequence identity
GT	Glycosyl transferase
GH	Glycoside hydrolase
AA	Auxiliary activity
CE	Carbohydrate esterase
CBM	Carbohydrate-binding module
