# Supplementary material for: Pan-Genome Analyses of Geobacillus spp. Reveal Genetic Characteristics and Composting Potential
Source: Int J Mol Sci. 2020 May 11;21(9):3393. doi: 10.3390/ijms21093393 (PMC7246994; doi:10.3390/ijms21093393)
Supplement: Supplementary file 1 [file ijms-21-03393-s001.zip › ijms-761044 for publication/Supplementary Figures.docx]

**Supplementary figure**

**Figure S1.** Pan– and core genome development plots of *G. stearothermophilus* (A), *G. thermocatenulatus* (B), *G. thermodenitrificans* (C) and *G. thermoglucosidasius* (D). The genome order was randomized ten times, and the random genome list was subjected to pan- and core genomes analyses. The boxplots of the pan- (cyan color) and core genomes (pink color) are plotted. The pan-genome represents the total genes present in the genomes of the included strains, and the core genome represents the genes shared by all genomes in the included strains.


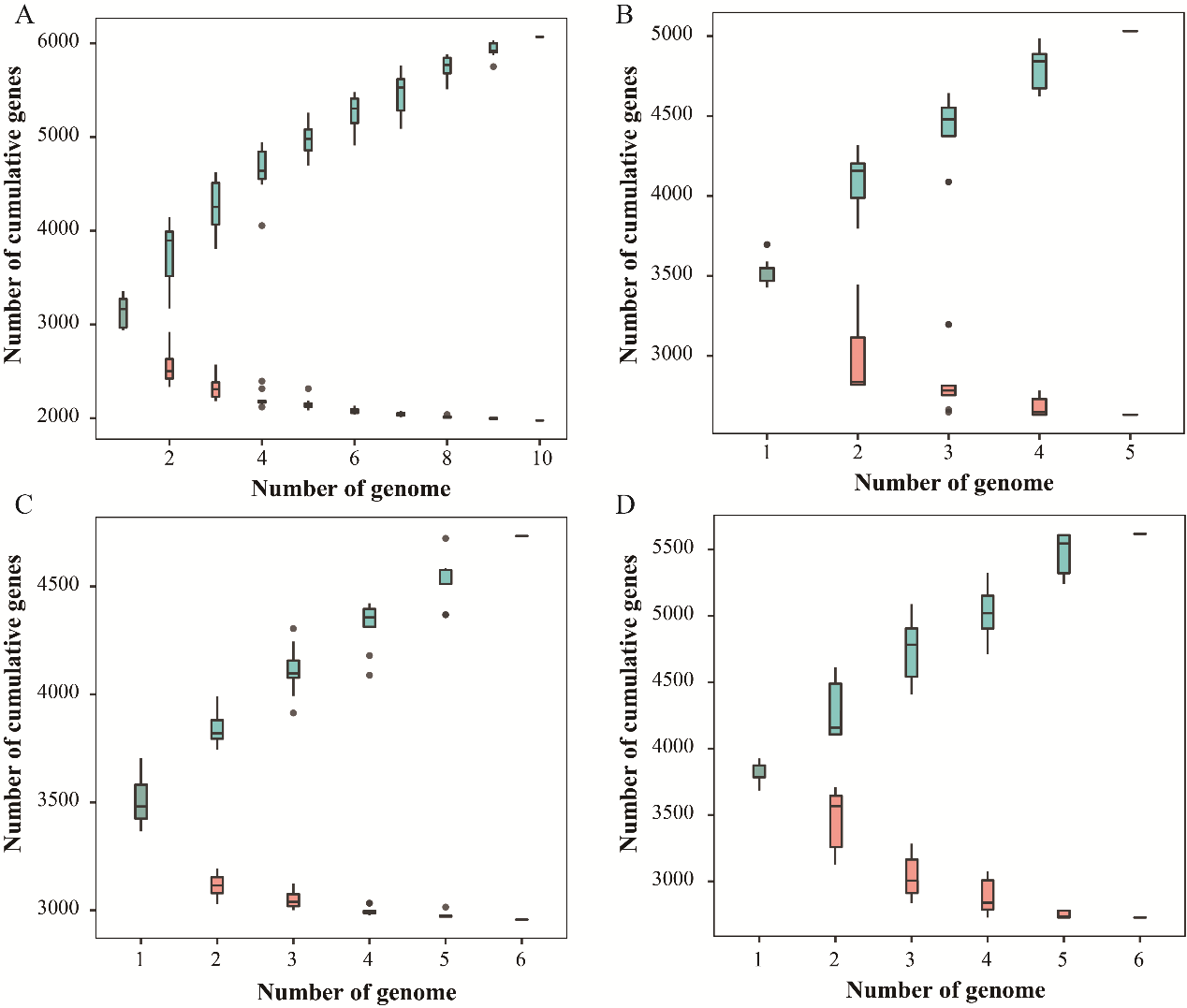


Figure. S1
